# Cutaneous tuberculosis—ambiguous transmission, bacterial diversity with biofilm formation in humoral abnormality: case report illustration

**DOI:** 10.3389/fpubh.2023.1091373

**Published:** 2023-04-21

**Authors:** Przemysław Zdziarski, Mariola Paściak, Anna Chudzik, Monika Kozińska, Ewa Augustynowicz-Kopeć, Andrzej Gamian

**Affiliations:** ^1^Department of Clinical Immunology, Tuberculosis and Pulmonary Disease, Lower Silesian Oncology Center, Wroclaw, Poland; ^2^Department of Immunology of Infectious Diseases, Hirszfeld Institute of Immunology and Experimental Therapy, Polish Academy of Sciences, Wroclaw, Poland; ^3^Department of Microbiology, National Tuberculosis and Lung Diseases Research Institute, Warsaw, Poland

**Keywords:** cutaneous tuberculosis, portal of entry/exit, Actinobacteria, microbiome, biofilm, paraproteinemia, diagnostic chain, translational medicine

## Abstract

**Background:**

Cutaneous tuberculosis (CTB) and its paucibacillary forms are rare and difficult to diagnose, especially in immunocompromised patients with significant comorbidity. The aim of the study was to introduce the modern concept of the microbiome and diagnostic chain into clinical practice (patient-centered care) with the presentation of an atypical form of cutaneous tuberculosis with necrotizing non-healing ulcers leading to polymicrobial infection.

**Methods:**

The study material included samples from sputum, broncho-alveolar lavage and skin ulcer, taken from a patient developing cutaneous tuberculosis. The microbiological investigation was performed, and identification of the isolates was carried out using genotyping and the matrix-assisted laser desorption ionization-time of flight mass spectrometry.

**Results:**

The immunocompromised patient with humoral abnormality (plasma cell dyscrasia) and severe paraproteinemia developed multiorgan tuberculosis. Although cutaneous manifestation preceded systemic and pulmonary symptoms (approximately half a year), the mycobacterial genotyping confirmed the same MTB strain existence in skin ulcers and the respiratory system. Therefore, the infectious chain: transmission, the portal of entry, and bacterial spreading *in vivo*, were unclear. Microbial diversity found in wound microbiota (among others *Gordonia bronchialis, Corynebacterium tuberculostearicum, Staphylococcus haemolyticus*, and *Pseudomonas oryzihabitans*) was associated with the spread of a skin lesion. The *in vitro* biofilm-forming capacity of strains isolated from the wound may represent the potential virulence of these strains. Thus, the role of polymicrobial biofilm may be crucial in ulcer formation and CTB manifestation.

**Conclusions:**

Severe wound healing as a unique biofilm-forming niche should be tested for Mycobacterium (on species and strain levels) and coexisting microorganisms using a wide range of microbiological techniques. In immunodeficient patients with non-typical CTB presentation, the chain of transmission and MTB spread is still an open issue for further research.

## 1. Introduction

Tuberculosis (TB) remains one of the world's major infectious diseases with a high impact on global health. Clinicians, especially in tropical countries, are confronted with a broad range of mycobacterial diseases with skin manifestations and the need for quick and accurate medical intervention (1). Cutaneous mycobacterial infections may cause a wide range of clinical manifestations, which are divided into four main disease categories: (i) cutaneous manifestations of *Mycobacterium tuberculosis* infection; (ii) Buruli ulcer (BU) caused by *Mycobacterium ulcerans* and other related slowly growing mycobacteria; (iii) leprosy caused by *Mycobacterium leprae* and *Mycobacterium lepromatosis*; and (iv) cutaneous infections caused by rapidly growing mycobacteria (2). Mycobacteria appear phenotypically most closely related to members of *Nocardia, Rhodococcus, Tsukamurella*, and *Corynebacterium* genera (3). *Mycobacterium tuberculosis* (MTB) is considered a predominantly airborne pathogen and the leading cause of death from a single infectious agent (4). CTB is reported as a local and single infectious agent disease (2). Although the coexistence of cutaneous and pulmonary tuberculosis is rare (5), historical descriptions of cutaneous tuberculosis (CTB) and presentation in dermatological atlases were based on the clinical manifestation (e.g., pulmonary symptoms), histopathology of skin lesions (Ziehl-Neelsen acid-fast stain (ZN), and morphology. However, the simple study does not resolve the basic doubts (e.g., a portal of entry or exit) as well as ZN stain technique does not exclude co-infection with other acid-fast bacteria (e.g., *Nocardia* spp., *Mycobacterium* spp., and other mycolata). Necrotizing skin ulcer positive for AFB by ZN staining was diagnosed and treated as BU, but interestingly cultivated strain belonged to the Euro-American Linage of *M. tuberculosis* (1). It indicates that the diagnostic methods established in the clinic are currently insufficient for effective therapeutic intervention. This approach requires basic elements of the diagnostic chain in microbiome analysis, recently described in autoimmune and humoral abnormality with Sjögren's syndrome (6). In this respect, matrix-assisted laser desorption ionization–time-of-flight mass spectrometry (MALDI-TOF MS) is a promising and increasingly available technique (7), used by microbiologists for microbial identification and has become a fast, favorable line of investigation in clinical diagnosis of polymicrobial diseases.

The present report introduces cutting-edge technologies in the diagnostics of infectious diseases. It includes a modern and comprehensive microbiome investigation (8) in multiorgan infectious processes on the example of cutaneous tuberculosis. It includes a wide-ranging microbiota investigation of CTB with an atypical presentation. The microbiota was determined by culture and molecular methods and analyzed by MALDI-TOF MS. The *in vitro* biofilm-forming capacity of strains isolated from the wound was evaluated. Moreover, an immunological evaluation of host factors was performed. The aim of the study was also to present the results of such an innovative approach demonstrating an atypical form of cutaneous tuberculosis with necrotizing non-healing ulcers leading to polymicrobial infection (dysbiosis). Thus, important methodological aspects for the diagnostics of CTB are underlined.

## 2. Materials and methods

### 2.1. Mycobacterial culture and detection

The samples from sputum, bronchoalveolar lavage (BAL), and skin ulcer were taken as previously described (6) and cultivated on solid Middlebrook and Lowenstein–Jensen medium at 30 and 37°C. Initial identification of *M. tuberculosis* complex was performed with MGIT TBc Identification Test (Becton Dickinson). Drug susceptibility testing was analyzed according to the WHO reference method (9). For *Mycobacterium tuberculosis* (MTB) identification, spoligotyping and mycobacterial interspersed a repetitive unit-variable number of tandem repetitive units (MIRU-VNTRs) typing were used (10). The standard 24 loci were performed for genotyping of MTB (11). To analyze the genetic relationship between the first (skin), second (sputum), and third (BAL) probes, the MIRU-VNTR plus application was implemented (10), and the multiorgan infection was defined as paired isolates with equivalent MIRU-VNTR patterns (12).

### 2.2. Culture conditions of skin ulcers microbiota

Two swabs taken from the skin ulcer were spread on solid media: blood agar, tryptic soy-thioglycollate agar (13), nutrient agar, and BHI (brain–heart infusion) agar and cultured in aerobic and anaerobic conditions. Aerobic cultures were obtained after 24 to 48 h at 37°C. Anaerobic conditions were obtained with the use of the GasPak ^TM^ EZ Anaerobe Container System after 4–7 days of incubation at 37°C. Pure colonies were selected and subjected to MALDI-TOF MS (matrix-assisted laser desorption ionization–time-of-flight mass spectrometry) analysis. Strains isolated from anaerobic culture conditions were additionally cultivated in aerobic conditions to check their oxygen sensitivity.

### 2.3. MALDI-TOF MS

The standard ethanol–formic acid protein extraction method was used according to the procedure recommended by the spectrometer manufacturer (14). MALDI-TOF MS analysis was performed with the Ultraflex mass spectrometer (Bruker Daltonics, Germany). The spectra were externally calibrated using the *E. coli* DH5-alpha standard (Bruker Daltonics). The Biotyper 3.1 software (Bruker Daltonics) with a database containing 8,469 entries was used for strain identification. The criteria used in identification were as follows: The logarithmic score value below 1.699 meant that the identification was unreliable, 1.7–1.999 probable genus identification, 2.0–2.299 reliable genus identification, and 2.3–3.0 highly probable species identification.

### 2.4. Biofilm-forming capacity measurements

The non-mycobacterial strains isolated from the wound were inoculated onto solid BHI media and incubated for 24 h (*E. cloacae, E. faecium, E. faecalis, S. hominis*, and *S. haemolyticus*) or 48 h for strains with a slower growth rate (*C. amycolatum, C. tuberculostearicum, G. bronchialis*, and *P. oryzihabitans*) in aerobic conditions. For comparison purposes, we used the biofilm-forming strain *S. xylosus* PCM 2122. Each strain was then suspended in BHI broth and additionally in TSB broth (*Corynebacterium* spp., *G. bronchialis*, and *P. oryzihabitans*) and incubated at 37°C. After obtaining an OD 600 equal to 0.2, 200 μl of bacterial suspension (six wells per one strain) was applied to 96-well flat-bottom plate (Nunclon^TM^ Delta Surface, Cat No. 167008, Denmark), covered with a lid, and incubated at 37°C under aerobic conditions for 24 h or 48 h (for slower growing strains). The procedure for measuring the biofilm-forming capacity was performed according to Skutlaberg et al. (15) with heat fixing and staining with crystal violet. A BioTek PowerWave XS microplate reader (BioTek, Winooski, VT, United States) was used for the OD600 measurements. Continuous variables are presented as the mean [95% confidence interval (CI)] and compared using one-way ANOVA. Analysis of variance was performed using Bartlett's test for equal variances and statistical significance using Bonferroni's multiple comparison tests for test strains vs. control. Statistical analyses were performed using GraphPad Prism 5.01.

## 3. Results

### 3.1. Multiorgan tuberculosis—Case illustration

**Figure 1:**
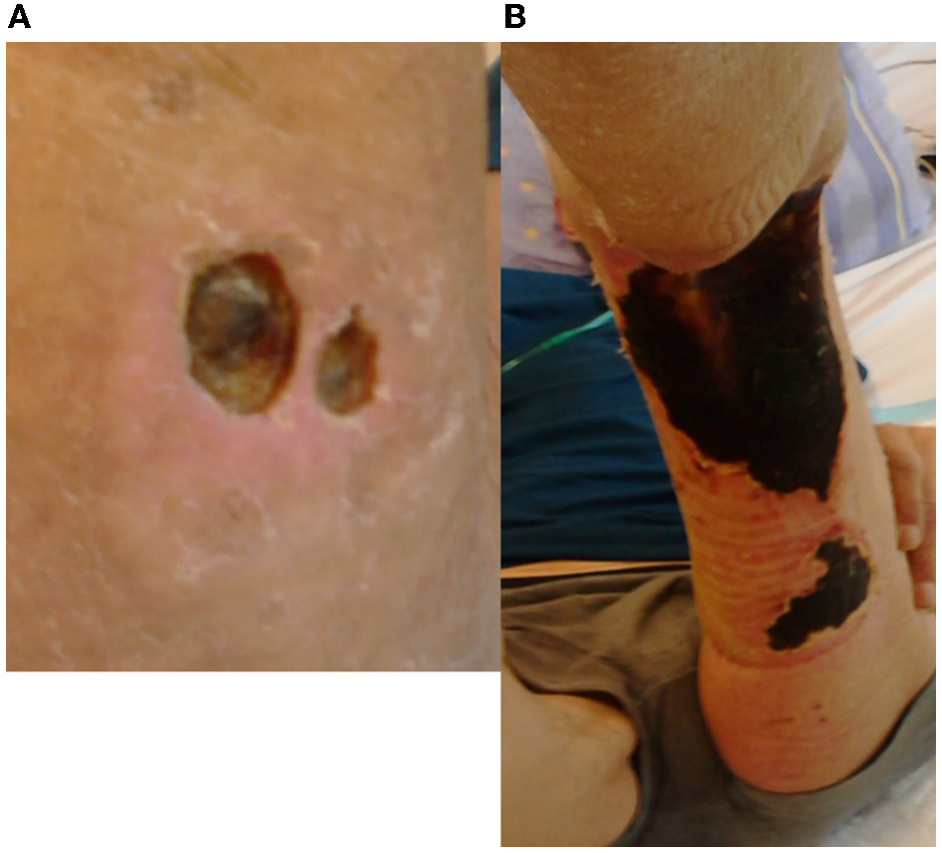
Clinical presentation of cutaneous tuberculosis as a necrotizing ulcer: **(A)** initial painless plaque and edema with ulceration; **(B)** developed diffuse ulcer. Unlike in scrofuloderma, enlarged lymph nodes were not observed in this case. Although asthenia and weight loss were observed, neither crusts nor plaques were observed. Deep ulcers cause scarring of muscles and tendons and finally permanent disability. Cloth necrosis but without purulent exudate was found **(A)**. The painless ulcer turned black (possibly due to dyscrasia, hyperviscosity, and hemolysis **(B)**. After the therapeutic regimen, the ulcer has not developed further.

A 64-year-old White man (non-smoker), with rheumatoid arthritis, recently diagnosed with plasma cell dyscrasias (i.e., light chain disease) with humoral abnormalities, was admitted to our hospital. Previously (~ half a year earlier) noticed ulcers appearing on the skin of the leg were initially diagnosed as a result of rheumatoid nodules. The patient did not use hot tubs, whirlpools, and swimming. According to the patient's account, the changes were initially non-specific, an unremarkable reddish papulonodular and painless lesion forms, which rapidly enlarged and erode within 1–2 months (Figure 1A). Despite treatment with topical glucocorticosteroids (3 months), then systemic [plus cyclophosphamide (100 mg/day)], the confluent lesions enlarged into big necrotizing ulcers. Subcutaneous nodules (symptoms of *P. aeruginosa* sepsis) were not observed (16). No purulent exudate, green color, or other signs of pseudomonas folliculitis were observed (Figure 1). Finally, enlarged non-healing demarcated ulceration (with an indurated base and edges) was initially red and then dark blue and finally turned black (Figure 1B) possibly due to hemolysis. Deep ulcers cause scarring of muscles and tendons, finally leading to permanent disability (Figure 1B). Unlike, in scrofuloderma, enlarged lymph nodes were not observed in this case. Although asthenia and weight loss have been observed, crusts and plaques were not seen. General swelling and a sensation of creeping on the skin were observed; next, renal and neurovascular (e.g., TIA) symptoms such as asthenia, bleeding, ocular, and cardiovascular manifestations appeared; loss of weight by 5 kg followed by pulmonary symptoms with multiple organ failure during admission to our hospital. There was no evidence of acute deep vein thrombosis of the lower limbs and saphenous venous thrombosis. Because of vasculopathy and the risk of hemorrhage (a constitutional symptom of hyperviscosity is bleeding and cardiovascular manifestations), surgical intervention and skin biopsy have not been performed. Contrary to the advancement of paraproteinemia, vascular process, and complications, respiratory symptoms did not dominate. Chest CT scan showed small thick-walled cavities that correspond with normal blood pressure, pH = 7.48, partial pressure of oxygen (pO2 51.4 mmHg), as well as lack of pulmonary symptoms, including dyspnea and cough. Noteworthy, other extrapulmonary lesions or miliary TB were excluded. A bronchoscopic examination was performed with BAL and sputum/skin sampling. After his TB diagnosis (see Section 2.1), he received a therapeutic regimen with conventional oral therapy (9, 17). The patient was treated with a combination of four chemotherapy drugs: rifampicin, ethambutol, pyrazinamide, and isoniazid (streptomycin was not considered due to nephrotoxicity). Due to the patient's condition (WHO4) and tuberculous complications, he was disqualified from further hemato-oncological treatment of plasma cell dyscrasia. Although the TB therapeutic regimen was introduced and the big skin ulcer and pulmonary symptoms were stable (no further enlargement of the necrotic ulcer was observed by 2 months), his health condition worsened day by day. The bacterial culture from blood (*Mycobacterium* spp. as well as aerobic and anaerobic bacteria) was negative. He died within 3 months after admission due to hypoalbuminemia, exudates, microcirculatory abnormalities, anuria (secondary membranous nephropathy), and finally brain edema and multiple organ insufficiency (there was no consent to the autopsy).

### 3.2. Host immune parameters

In the presented case, the cutaneous manifestation as an ulcer (Figure 1A) preceded pulmonary symptoms. In the initial period of skin ulcer and progression of rheumatological disease in qualifying for immunotherapy, the crucial TB tests, such as X-ray and QuantiFERON (QuantiFERON-TB Gold Plus^®^, QIAGEN), were negative. Humoral disorder and paraproteinemia were observed but no lymphopenia or cellular disorders. Primary immunodeficiency was excluded in the patient but profound secondary humoral abnormalities were observed. Very high β_2_-microglobulin level (5.38 mg/L, normal value 1.03–2.58) and lactate dehydrogenase (LDH) level (555 U/L; 0–248) corresponded to severe plasma cell dyscrasia, paraproteinemia, diminished blood circulation, and hypoxia. Stage 3 (Revised International Staging System for multiple myeloma) and severe hypoalbuminemia were the main causes of the patient's deteriorating condition (vascular and kidney damage, exudates). Interestingly, the patient did not show cellular immunodeficiency with a typical collection of opportunistic fungal and viral pathogens (the patient was HIV-negative). Although CRP was elevated (as observed in Stage 3 plasma cell dyscrasia, i.e., 169 mg/L), procalcitonin was insignificant at 0.00148 mg/L.

### 3.3. Mycobacterium tuberculosis identification

Microscopic examination of fine-needle aspirates from an atypical non-healing wound showed necrosis and inflammation without acid-fast bacilli, which are observed in spontaneous sputum (Table 1). Mycobacterial culture performed on the Middlebrook and Lowenstein-Jensen medium at 37°C took 5 and 7 weeks, respectively (four bacterial colonies), unlike the sputum specimen with abundant growth (3 weeks). Mycobacterial cultures performed on the same solid media at 30°C, recommended for *M*. *ulcerans*, were negative. Initial identification of MTB complex from both specimens was confirmed with MGIT TBc Identification Test^®^ (Becton Dickinson) (Table 1). Strict MTB identification, spoligotyping, and mycobacterial interspersed a repetitive unit-variable number of tandem repetitive units (MIRU-VNTRs) typing revealed that ulcer-associated *Mycobacterium tuberculosis* (MTB) had the same genetic profile as that isolated from the respiratory system (Table 1) but a different nature of growth under influence different niche.

**Table 1:** Comparison of two mycobacterial isolates (the same strain) from extrapulmonary (skin ulcer) and respiratory (sputum/BAL) niches.

	**Paucibacillary skin ulcer**	**Sputum/BAL**
Onset of symptoms	March/April	About 4–8 weeks later
Middlebrook's medium	Positive (5 weeks)	Positive (3 weeks)
Lowenstein-Jensen's medium	Weak growth−4 bacterial colonies (7 weeks)	Multibacillary growth
Acid-fast bacilli	Negative	Positive
Other microbial species^a^	Abundant	Predominantly oral origin (*Streptococcus orale, Neisseria* spp., *Candida* spp.) BAL(-)
Cytology	Inflammatory with granulocytes	Predominantly epithelial cells
Initial identification^b^	*Mycobacterium tuberculosis* complex	*Mycobacterium tuberculosis* complex
Drug susceptibility testing	sensitive to all anti-TB drugs	sensitive to all anti-TB drugs
MTB Spoligotyping	Lineage T1, ST n° 53	Lineage T1, ST n0 53
MIRU-VNTRs	333633242232225	333633242232225

^a^Microbiota isolated from paucibacillary skin ulcer is shown in Table 2.

^b^Initial identification of *M. tuberculosis* (MTB) complex from both specimens was performed with MGIT TBc Identification Test (Becton Dickinson) and positive niacin test.

### 3.4. Biofilm-forming species identification

**Figure 2:**
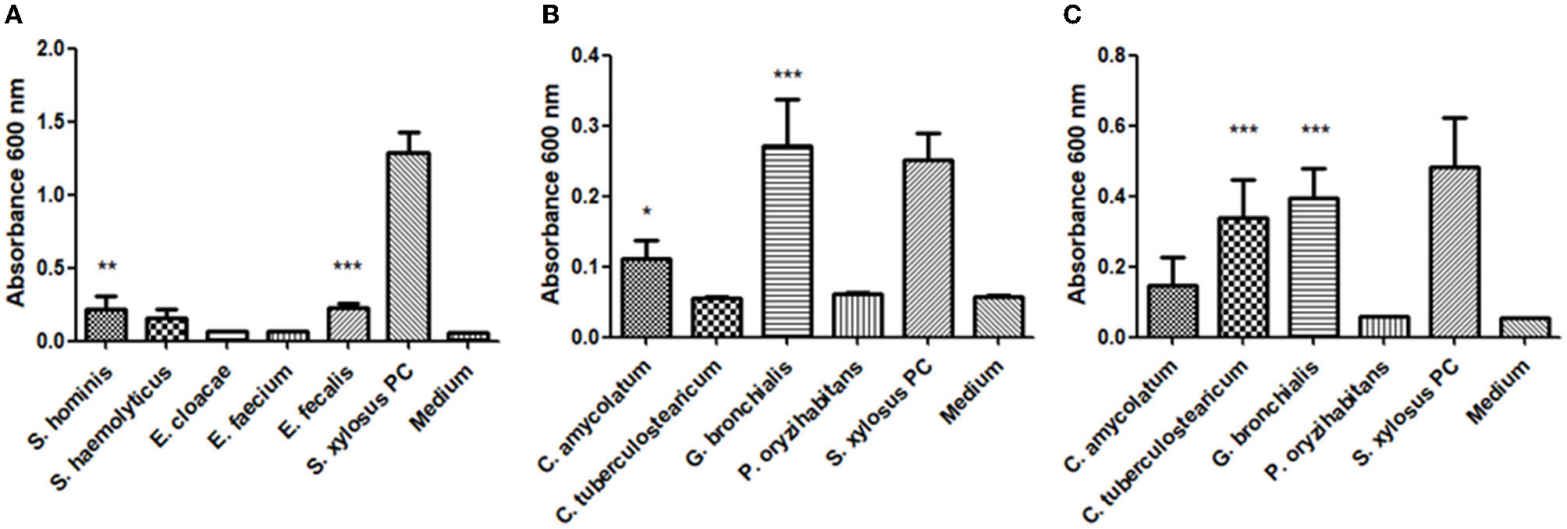
The biofilm-forming capacity of strains isolated from a necrotizing ulcer: **(A)** *S. hominis, S. haemolyticus, E. cloacae, E. faecium, E. faecalis, and S. xylosus* PCM 2122 grown in BHI broth for 24 h; **(B)** *C. amycolatum, C. tuberculostearicum, G. bronchialis, P. oryzihabitans, and S. xylosus* PCM 2122 grown in BHI broth for 48 h; **(C)** *C. amycolatum, C. tuberculostearicum, G. bronchialis, P. oryzihabitans, and S. xylosus* PCM 2122 grown in TSB broth for 48 h. Statistical significance was calculated using a one-way analysis of ANOVA, followed by Bonferroni's multiple comparison test (*p < 0.05, **p < 0.01, and ***p < 0.001).

Microorganisms cultured from the skin ulcer in anaerobic (generated in Gas Pack^TM^) and aerobic conditions were isolated to form pure colonies and analyzed by MALDI-TOF Biotyper (Table 2). Interestingly, in the aerobic conditions, the culture presented a narrower microbial collection and fewer bacterial species were identified (Table 2). The skin ulcer aerobic culture after 24 h revealed *Enterobacter cloacae* and *Staphylococcus haemolyticus*. Anaerobic culture after 4 days of incubation revealed more bacterial species. It is important to also mention that all species isolated initially from anaerobic conditions grew well also in aerobic conditions. Cultures for bacteria, *Candida* spp., dermatophytes, and other fungal pathogens in the BAL sample were negative. Due to the different growth rates of the isolates, the biofilm-forming capacity test was performed separately for 24 h and 48 h for fast- and slow-growing species, respectively. From nine strains cultivated in BHI broth, we found four isolates with moderate biofilm-forming capacity, that is, *S. hominis, E. faecalis, C. amycolatum*, and *G. bronchialis* (Figures 2A, B). However, we found that *C. tuberculostearicum* did not grow in this medium under test conditions, and the test was repeated in TSB broth. In the TSB broth medium, the *C. tuberculostearicum* has a biofilm-forming capacity similar to *G. bronchialis* and contrary to *C. amycolatum* (Figure 2C).

**Table 2:** Non-mycobacterial microbiota isolated from a necrotizing ulcer.

**Organisms identification**	**Growth condition^a^**	**Score value^b^**
*Corynebacterium amycolatum*	Aerobic/Anaerobic	2.120/2.044
*Corynebacterium tuberculostearicum*	Anaerobic	2.035
*Enterobacter cloacae*	Aerobic/Anaerobic	2.451/2.396
*Enterococcus faecalis*	Anaerobic	2.472
*Enterococcus faecium*	Anaerobic	2.120
*Gordonia bronchialis*	Anaerobic	2.101
*Pseudomonas oryzihabitans*	Anaerobic	2.350
*Staphylococcus epidermidis*	Anaerobic	1.749
*Staphylococcus haemolyticus*	Aerobic/Anaerobic	2.189/2.040
*Staphylococcus hominis*	Anaerobic	2.002

^a^Condition of strain isolation; anaerobic atmosphere generated in Gas Pack^TM^.

^b^Log score values determined by MALDI Biotyper: < 1.7—identification not reliable, 1.7–2.0—probable genus identification, 2.0–2.3—secure genus identification and probable species identification, and > 2.3—highly probable species identification.

## 4. Discussion

A clinical paradigm is that MTB is transmitted by airborne transmission. Indeed, the main route of transmission of TB is the air (95% of cases) through droplets, and digestive transmission (potentially requires a higher concentration of bacilli) is less frequently reported because of methodological difficulties and classification. Cutaneous transmission is also a possible route of infection, especially among medical personnel. However, rare cases of community-acquired cutaneous tuberculosis, presented in Europe as historical, were also described as exogenous infections (18, 19). In the presented case, we proved that the same MTB strain was isolated from the skin ulcer and the sputum by using molecular typing (Table 1). The patient presented skin lesions much earlier than pulmonary symptoms, but our analysis did not allow us to clearly state whether there was simultaneous exposure or bloodborne spread of MTB (skin as a portal of entry or exit). Interestingly, no other visceral/extra-thoracic (e.g., intestinal or kidney) tuberculosis was found. The patient showed no typical risk factors for exogenous inoculation of mycobacteria such as circumcision, jail-house tattooing, or piercing. The manifestation resembled tuberculous chancre (an exogenous form of CTB) that occurs in patients who are non-sensitized (17, 19). Furthermore, contrary to numerous descriptions of CTB, the skin lesions presented here are not typical of any form of CTB (20). In our patient, the extent of lesion and ulceration suggested a different species (e.g., *M. ulcerans*); however, it may be also the result of decreased immunity or healing disorders.

A similar case initially diagnosed and treated as Buruli ulcer (BU) was previously reported; however, no host immunodiagnostic testing and pulmonary specimen analysis were performed (1). Noteworthy, the coincidence of pulmonary and CTB has not been analyzed comprehensively. Reports of CTB without baseline pulmonary manifestation are common in the literature. Boreta and Green showed comparable CTB cases without acid-fast bacilli in punch biopsy, positive results of MTB culture but without sputum analysis and pulmonary presentation (21). Olteanu et al. (22) presented a pulmonary tuberculosis case complicated with laryngeal tuberculosis and “cutaneous tuberculosis” but confirmed by histopathological examinations only without species identification. Only one previous study showed the coexistence of both forms at a low level (of 370 patients, only 13 (3.51%) has CTB) but confirmed based on histology only. The authors did not determine whether the same species and strain exist in the skin and the lung (5). To the best of our knowledge, our case is the first confirmation of the same MTB strains isolated from two organs by molecular methods. It is important for public health and pharmacotherapy because species identification in CTB may be crucial as an example of *M. bovis* belonging to the *M. tuberculosis* complex, which is innately resistant to pyrazinamide.

Cutaneous tuberculosis remains one of the least studied and often under-reported variants of extrapulmonary TB because of its wide and variable clinical presentation. Knowing that every stage of cutaneous mycobacterial disease development resembles clinical features of other diseases (e.g., pyoderma gangrenosum, pseudomonas folliculitis, earlier differentiated), especially on the lower limbs, this approach runs the risk that a wrong diagnosis is made (23). Now strict diagnosis requires sensitive and specific microbiological confirmation (not AFB smear only) with a wide repertoire of various techniques (genomics, culturomics, etc., see Tables 1, 2; Figure 2) (8) in the standardized diagnostic chain in clinical practice (6). Such analysis and well-developed concept are fundamentals for translational medicine and patient-centered care in humoral immunodeficiency and plasma cell abnormalities (24–26).

Cutaneous tuberculosis is more difficult to diagnose and therapy than its pulmonary form because there are other competing biofilm-forming bacteria as presented in the Buruli ulcer case (27) and there is limited access to oxygen (28). Only some microorganisms in the specific niche will survive competition for oxygen, nutrients (low-nutrient environments), and space, especially in the context of host factors. Thus, the biofilm is one of the key elements of the microbiome for the clinical manifestation of CTB, but this has not been studied extensively. Mycobacteria by their hydrophobic outer membrane and multiple adaptations are resistant to desiccation, acids, and the host immune system (29). The difficulty in treating these infections could be due to the fact that biofilm is a well-established mechanism of antibiotic resistance to *M. tuberculosis* (as reviewed by 28). However, the role of biofilms in the pathogenesis of tuberculosis remains unclear, but participation in the process of caseous necrosis and cavitation formation in lung tissue is proposed (30). Our observation of host factor and microbiota (*Gordonia bronchialis* and multiresistant *Corynebacterium tuberculostearicum*, Table 2) in CTB may be a new observation: Destruction of cutaneous tissues and creation of a unique niche in the skin promotes skin adhesion, survival, and CTB progress (Figure 1).

First, the hydrophobic properties were observed in patient hyperviscosity, caused by severe paraproteinemia and high γ-globulin levels (see Section 3.2). Noteworthy, hypoalbuminemia increases viscosity by decreasing red cell deformability and increasing serum fibrinogen, observed in our patient (data not shown), bleeding, constitutional symptoms, ocular, neurological, and cardiovascular manifestations.

Second, among ulcer microbiota, we found Corynebacterium tuberculostearicum an emerging pathogen—also lipophilic, and usually a multiresistant strain (31). It is worth mentioning that *C. tuberculostearicum* was first time isolated from a leprosy patient in 1984 (32). It is worth noting that only three species were isolated from both aerobic and anaerobic cultures (Table 2), and paucibacillary growth in skin ulcers (Table 1) may be caused by limited oxygen access. One study showed that the anaerobic growth of MTB could be observed only when microaerobic-grown colonies were subcultured under anaerobic conditions (28). Therefore, our observation of anaerobic growth predominance (Table 2) suggests that biofilm is the adaptation to the reduced oxygen tensions and MTB can grow under micro- or anaerobic conditions. Moreover, *Gordonia bronchialis*, another example of Actinobacteria, has never been reported in CTB wounds. Notably, these acid-fast bacteria were isolated from the sputum of patients with cavitary tuberculosis and bronchiectasis (33, 34) or after medical procedures (35). Thus, *G. bronchialis* and other representatives of the order *Mycobacteriales* grown in a unique niche (Figure 1) and biofilm formation may be crucial for tuberculosis presentation (Table 2) (33). The coexistence of both acid-fast bacteria in different localizations is probably not a random finding, but it requires further research with various microbiological techniques and co-cultivation (culturomics) (8). Moreover, the biofilm-forming capacity found in over half of the studied strains seems to be important in wound colonization and competition between different species (36).

In the current treatment of CTB, there are no registered topical drugs, and orally administered drugs cannot reach superficial foci, especially poorly vascularized ones. Even though we detected the same strain in the BAL and skin, it is modified by cutaneous biofilm (Table 1). Therefore, the treatment can be difficult as biofilm per se limits pharmacokinetics (30). Many CTB guidelines omit them altogether [e.g., Canadian Tuberculosis Standards 7th Edition—Nonrespiratory Tuberculosis (37)].

Our study has a few limitations. First, it is a single and rare case of CTB, which has not been reported in Europe in a long time and has not been described in Poland in the last decades. It is, therefore, difficult to arrange a cohort study, since the patient did not belong to the high-risk group of T-cell deficiency. It is important to note that in official statistics (WHO and ECDC), any bacteriologically confirmed or clinically diagnosed case of TB involving organs or anatomical sites other than the lungs was collectively reported as extrapulmonary TB (retrospective analysis of CTB and pulmonary TB coincidence is difficult). Second, in the clinical course, there are no clear data on when and how pulmonary primary lesions spread to the skin (or vice versa). Thus far, however, it has been assumed as a paradigm that there is a bloodborne transmission, although simultaneous exposure of the skin and lungs may have occurred, especially when there was no species identification of the MTB complex in literature.

In conclusion, severe non-healing, necrotizing/ulcerative wounds and vascular disease as a unique biofilm-forming niche should be tested for *Mycobacterium* (on species and strain levels) and coexisting microorganisms using a wide range of microbiological techniques. In the polymicrobial cutaneous infection, *M. tuberculosis* may be overlooked, because of non-selective bacterial cultures. In patients with immunodeficiency and high comorbidity and non-typical presentation, the chain of transmission and MTB spread in CTB is still an open issue and an open field for future research and translational medicine.

## Data availability statement

The datasets presented in this study can be found in online repositories. The names of the repository/repositories and accession number(s) can be found below: All data generated or analyzed during this study are included in this article. The bacterial isolates were deposited in the Polish Collection of Microorganisms (Hirszfeld Institute of Immunology and Experimental Therapy, Wroclaw, Poland), mycobacterial strains – in National Tuberculosis and Lung Diseases Research Institute, Warsaw, Poland. Clinical isolates: Enterobacter cloacae PCM 3192, Enterococcus faecalis PCM 3194, Enterococcus faecium PCM 3195, Staphylococcus hominis PCM 3196, Staphylococcus haemolyticus PCM 3197, Gordonia bronchialis PCM 3198, Pseudomonas oryzihabitans PCM 2199, Corynebacterium amycolatum PCM 3193 and Corynebacterium tuberculostearicum PCM 2200.

## Ethics statement

Ethical review and approval was not required for the study on human participants in accordance with the local legislation and institutional requirements. The patients/participants provided their written informed consent to participate in this study.

## Author contributions

PZ and MP conceived and designed the experiments. PZ was responsible for the clinical diagnosis and patient management. MP and AC performed the microbiological, biofilm, and MALDI-TOF MS analysis. PZ, MP, and AG analyzed the data. AG contributed reagents/materials/analysis tools. MK performed the *Mycobacterium tuberculosis* spoligotyping and MIRU-VNTRs typing. EA-K analyzed the mycobacterial data. PZ wrote the manuscript. EA-K, MP, and AG reviewed and edited the manuscript. All authors read and approved the final manuscript.

## References

[1] Bratschi MW Njih Tabah E Bolz M Stucki D Borrell S Gagneux S. A case of cutaneous tuberculosis in a buruli ulcer–endemic area. PLOS Negl Trop Dis. (2012) 6:e1751. 10.1371/journal.pntd.0001751 22953005 PMC3429378

[2] Franco-Paredes C Marcos LA Henao-Martínez AF Rodríguez-Morales AJ Villamil-Gómez WE Gotuzzo E. Cutaneous mycobacterial infections. Clin Microbiol Rev. (2018) 32:e00069–18. 10.1128/CMR.00069-18 30429139 PMC6302357

[3] Gupta RS. Commentary: genome-based taxonomic classification of the phylum actinobacteria. Front Microbiol10. (2019) 10: 206. 10.3389/fmicb.2019.00206 30853945 PMC6395429

[4] WHO/ECDC. (2021). Tuberculosis surveillance and monitoring in Europe (2019 data)

[5] Kivanç-Altunay I Baysal Z Ekmekçi TR Köslü A. Incidence of cutaneous tuberculosis in patients with organ tuberculosis. Int. J. Dermatol. (2003) 42:197–200. 10.1046/j.1365-4362.2003.01762.x 12653914

[6] Zdziarski P Paściak M Gamian A. Microbiome analysis and pharmacovigilance after inhaled glucocorticoid: oral dysbiosis with the isolation of three rothia species and subsequent sjögren's syndrome. Front Pharmacol. (2022) 1:636180. 10.3389/fphar.2022.636180 35431920 PMC9010876

[7] Singhal N Kumar M Kanaujia PK Virdi JS. MALDI-TOF mass spectrometry: an emerging technology for microbial identification and diagnosis. Front Microbiol. (2015) 6:791. 10.3389/fmicb.2015.00791 26300860 PMC4525378

[8] Berg G Rybakova D Fischer D Cernava T Vergès MC Charles T. Microbiome definition re-visited: old concepts and new challenges. Microbiome. (2020) 8:103. 10.1186/s40168-020-00875-0 32605663 PMC7329523

[9] WHO. (2009). Guidelines for Surveillance of Drug Resistance in TB WHO 4th version. Geneva: World Health Organization.

[10] Augustynowicz-Kopeć E Jagielski T Kozińska M Kremer K van Soolingen D Bielecki J. Transmission of tuberculosis within family-households. J Infect. (2012) 64:596–608. 10.1016/j.jinf.2011.12.022 22327051

[11] Supply P Allix C Lesjean S Cardoso-Oelemann M Rüsch-Gerdes S Willery E. Proposal for standardization of optimized mycobacterial interspersed repetitive unit-variable-number tandem repeat typing of *Mycobacterium tuberculosis*. J Clin Microbiol. (2006) 44:4498–510. 10.1128/JCM.01392-06 17005759 PMC1698431

[12] Shao Y Song H Li G Li Y Li Y Zhu L. Relapse or re-infection, the situation of recurrent tuberculosis in eastern China. Front. Cell Infect. Microbiol. (2021) 17:11.638990. 10.3389/fcimb.2021.638990 33816342 PMC8010194

[13] Pasciak M Holst O Lindner B Mordarska H Gamian A. Novel bacterial polar lipids containing ether-linked alkyl chains, the structures and biological properties of the four major glycolipids from Propionibacterium propionicum PCM 2431 (ATCC 14157T). J. Biol. Chem. (2003) 7:278.3948–56. 10.1074/jbc.M206013200 12427753

[14] Paściak M Dacko W Sikora J Gurlaga D Pawlik K Miekisiak G. Creation of an in-house matrix-assisted laser desorption ionization-time of flight mass spectrometry corynebacterineae database overcomes difficulties in identification of nocardia farcinica clinical isolates. J Clin Microbiol. (2015) 53:2611–21. 10.1128/JCM.00268-15 26041903 PMC4508453

[15] Skutlaberg DH Wiker HG Mylvaganam H Norrby-Teglund A Skrede S INFECT Study Group. (2022). Consistent biofilm formation by streptococcus pyogenes emm 1 isolated from patients with necrotizing soft tissue infections. Front. Microbiol. 18, 13.822243. 10.3389/fmicb.2022.822243 35250938 PMC8895234

[16] Wu DC Chan WW Metelitsa AI Fiorillo L Lin AN. Pseudomonas skin infection: clinical features, epidemiology, and management. Am. J. Clin. Dermatol. (2011) 1:12.157–69. 10.2165/11539770-000000000-00000 21469761

[17] van Zyl L du Plessis J Viljoen J. Cutaneous tuberculosis overview and current treatment regimens. Tuberculosis. (2015) 95:629–38. 10.1016/j.tube.2014.12.006 26616847

[18] Frankel A Penrose C Emer J. Cutaneous tuberculosis: a practical case report and review for the dermatologist. J Clin Aesthet Dermatol. (2009) 2:19–27. 20725570 PMC2923933

[19] Barbagallo J Tager P Ingleton R Hirsch RJ Weinberg JM. Cutaneous tuberculosis: diagnosis and treatment. Am J Clin Dermatol. (2002) 3:319–28. 10.2165/00128071-200203050-00004 12069638

[20] Tanaka A Kato Y Arai K Oh-i T Koga M. Unusual clinical features of cutaneous tuberculosis in an immune compromised patient. J. Dermatol. (2002) 29:226–31. 10.1111/j.1346-8138.2002.tb00254.x 12027088

[21] Borretta L Green P. An atypical presentation of lupus vulgaris. CMAJ. (2017) 189:E469. 10.1503/cmaj.160148 28385863 PMC5367994

[22] Olteanu M Popescu MR Nitu M Calarasu C Maceseanu AV Olteanu M. Rare case of pulmonary tuberculosis with hematogenous spread to larynx and skin. Curr Health Sci J. (2016) 42:213–6. 10.12865/CHSJ.42.02.15 30568835 PMC6256154

[23] Van Leuvenhaege C Vandelannoote K Affolabi D Portaels F Sopoh G de Jong BC. (2017). Bacterial diversity in Buruli ulcer skin lesions: challenges in the clinical microbiome analysis of a skin disease. PLoS ONE 12:e0181994. 10.1371/journal.pone.0181994 28750103 PMC5531519

[24] Zdziarski P Wzorek A. Respiratory microbiota as a good predictor of outcome in humoral immunodeficiency. Methodological and conceptual difficulties in comprehensive microbiome analysis. Pol Med J. (2022)50:391–3. 36283010

[25] Zdziarski P Paściak M Rogala K Korzeniowska-Kowal A Gamian A. Elizabethkingia miricola as an opportunistic oral pathogen associated with superinfectious complication in humoral immunodeficiency: a case report. BMC Infect. Dis. (2017) 17:763. 10.1186/s12879-017-2886-7 29233117 PMC5727958

[26] Zdziarski P. Importance of the tumor boards' decisions for the patient-centered care-case studies in the COVID-19 era. Pol. Merkur. Lekarski. (2022) 19:50.78–85. 35436268

[27] Marsollier L Brodin P Jackson M Korduláková J Tafelmeyer P Carbonnelle E. Impact of Mycobacterium ulcerans biofilm on transmissibility to ecological niches and Buruli ulcer pathogenesis. PLoS Pathog. (2007) 3:e62. 10.1371/journal.ppat.0030062 17480118 PMC1864991

[28] Udou T. Adaptation of mycobacteria on solid, egg-based media to anaerobic conditions and characterization of their diagnostic phenotypes. J UOEH. (2013) 1:35. 10.7888/juoeh.35.109 23774654

[29] Grzegorzewicz A Paściak M. The key factors contributing to the risk, diagnosis and treatment of non-tuberculous mycobacterial opportunistic infections. Adv Hyg Exp Med. (2021) 75:696–710. 10.2478/ahem-2021-0050

[30] Esteban J García-Coca M. Mycobacterium biofilms. Front Microbiol. (2018) 8:2651. 10.3389/fmicb.2017.02651 29403446 PMC5778855

[31] Hinic V Lang C Weisser M Straub C Frei R Goldenberger D. Corynebacterium tuberculostearicum: a potentially misidentified and multiresistant Corynebacterium species isolated from clinical specimens. J Clin Microbiol. (2012) 50:2561–7. 10.1128/JCM.00386-12 22593594 PMC3421492

[32] Brown S Lanéelle MA Asselineau J Barksdale L. Description of Corynebacterium tuberculostearicum sp. *nov.*, a leprosy-derived *Corynebacterium*. Ann. Microbiol. (1984) 135B: 251–267. 10.1016/S0769-2609(84)80093-9 6532280

[33] Tsukamura M. Proposal of a new genus, Gordonia, for slightly acid-fast organisms occurring in sputa of patients with pulmonary disease and in soil. J Gen Microbiol. (1971) 68:15–26. 10.1099/00221287-68-1-15 4109926

[34] Savini V Fazii P Favaro M Astolfi D Polilli E Pompilio A. Tuberculosis-like pneumonias by the aerobic actinomycetes Rhodococcus, Tsukamurella and Gordonia. Microbes Infect. (2012) 14:401–10. 10.1016/j.micinf.2011.11.014 22192786

[35] Bartolomé-Álvarez J Sáez-Nieto JA Escudero-Jiménez A Barba-Rodríguez N Galán-Ros J Carrasco G. Cutaneous abscess due to Gordonia bronchialis: case report and literature review. Rev Esp Quimioter. (2016) 29:170–3. 27015823

[36] Płusa T. The importance of biofilm in the context of increasing bacterial resistance to antibiotics. Pol Merkur Lekarski. (2019) 47:197–202. 31812976

[37] Canadian Tuberculosis Standards 7th Edition. Available online at: https://www.canada.ca/en/public-health/services/infectious-diseases/canadian-tuberculosis-standards-7th-edition.html (accessed March 23, 2020).

